# Molecular and Pigment Analyses Provide Comparative Results When Reconstructing Historic Cyanobacterial Abundances from Lake Sediment Cores

**DOI:** 10.3390/microorganisms10020279

**Published:** 2022-01-25

**Authors:** Maïlys Picard, Susanna A. Wood, Xavier Pochon, Marcus J. Vandergoes, Lizette Reyes, Jamie D. Howarth, Ian Hawes, Jonathan Puddick

**Affiliations:** 1 Cawthron Institute, Private Bag 2, Nelson 7042, New Zealand; susie.wood@cawthron.org.nz (S.A.W.); xavier.pochon@cawthron.org.nz (X.P.); jonathan.puddick@cawthron.org.nz (J.P.); 2 Department of Biological Sciences, School of Biological Sciences, University of Waikato, Hamilton 3216, New Zealand; ian.hawes@waikato.ac.nz; 3 Institute of Marine Science, University of Auckland, Private Bag 349, Auckland 0941, New Zealand; 4 GNS Science, 1 Fairway Drive, Avalon, Lower Hutt 5011, New Zealand; m.vandergoes@gns.cri.nz (M.J.V.); l.reyes@gns.cri.nz (L.R.); 5 School of Geography, Environment and Earth Sciences, Victoria University of Wellington, P.O. Box 600, Wellington 6012, New Zealand; jamie.howarth@vuw.ac.nz

**Keywords:** 16S rRNA gene, droplet digital PCR, high-performance liquid chromatography, sedimentary DNA, cyanopigments, paleolimnology

## Abstract

Understanding the historical onset of cyanobacterial blooms in freshwater bodies can help identify their potential drivers. Lake sediments are historical archives, containing information on what has occurred in and around lakes over time. Paleolimnology explores these records using a variety of techniques, but choosing the most appropriate method can be challenging. We compared results obtained from a droplet digital PCR assay targeting a cyanobacterial-specific region of the 16S rRNA gene in sedimentary DNA and cyanobacterial pigments (canthaxanthin, echinenone, myxoxanthophyll and zeaxanthin) analysed using high-performance liquid chromatography in four sediment cores. There were strong positive relationships between the 16S rRNA gene copy concentrations and individual pigment concentrations, but relationships differed among lakes and sediment core depths within lakes. The relationships were more consistent when all pigments were summed, which we attribute to different cyanobacteria species, in different lakes, at different times producing different suites of pigments. Each method had benefits and limitations, which should be taken into consideration during method selection and when interpreting paleolimnological data. We recommend this biphasic approach when making inferences about changes in the entire cyanobacterial community because they yielded complementary information. Our results support the view that molecular methods can yield results similar to traditional paleolimnological proxies when caveats are adequately addressed.

## 1. Introduction

Information about past events, such as historical weather trends and the effects of anthropogenic actions, can provide valuable insights to understand the present and to predict the future [1,2,3,4,5,6]. To learn about past events that happened in and around lakes, paleolimnology has traditionally examined physical remains preserved in lake sediments [7]. These proxies are based on the resistant physical parts of some organisms such as insect mouthparts, pollen and spores from plants, and diatom frustules. Pigments have also been used for decades to retrace historical patterns in photosynthetic communities and can target a wide range of organisms [8,9,10,11,12], but they do not allow species-specific identification in the way that diatom frustules do. This used to be troublesome when studying photosynthetic communities which do not leave morphological remains behind when they die, such as cyanobacteria.

Cyanobacteria are photosynthetic prokaryotes that have inhabited Earth for an estimated three billion years [13]. They have recently received increasing scientific and public attention due to the impact of their excessive proliferations (blooms), which degrade aquatic ecosystem health and often produce life-threatening toxins [14,15,16,17,18,19]. Reports indicate that cyanobacterial blooms are increasing in frequency and magnitude in many waterbodies around the globe. Due to a lack of long-term monitoring records, there is uncertainty as to whether there is an actual rise in blooms or if an increase in awareness is leading to more reports [20]. In the case of the former, it is important to be able to pinpoint what triggered this increase, which can be evaluated using paleolimnology. However, unlike diatoms, cyanobacteria have soft cell walls, which degrade quickly once the cell dies; therefore, the only proxies available to study them in sediment cores are the molecules they produce. Studies to date have targeted their pigments [10,20,21,22,23,24,25], toxins (anatoxin, microcystin [24,26]), lipid biomarkers (e.g., 2-methylbacteriohopanetetrol as a possible biomarker for a freshwater strain of *Synechococcus* [27]), and now, their DNA [22,24,27,28,29,30].

Recently, molecular analysis of sedimentary DNA (sedDNA, intra- and extra-cellular DNA from bulk sediment samples) has been applied to paleolimnological studies [31]. These methods can target a range of organisms across a broad spectrum of taxa, with high levels of specificity and sensitivity [32,33,34]. However, limited comparisons of sedDNA with traditional paleolimnological proxies (such as pigments for cyanobacteria) have been made so far. One study did reconstruct total cyanobacterial abundances using quantitative PCR (qPCR, targeting a cyanobacterial specific 16S ribosomal RNA (16S rRNA) gene) from the sediment cores of five lakes in Western Quebec (Canada), and compared them to two pigments found in cyanobacteria (echinenone and zeaxanthin) [22]. However, when present, the pigments were not always correlated significantly and/or positively with total cyanobacteria abundance. In another study focusing on the history of cyanobacterial blooms in Anderson Lake (Washington State, USA), cyanobacterial pigments were quantified (specifically echinenone, zeaxanthin, canthaxanthin and myxoxanthophyll) and cyanobacterial sedDNA abundances measured using qPCR (cyanobacterial-specific 16S rRNA gene) [24]. No direct comparisons were made, however, their results showed an exponential increase in cyanobacterial sedDNA abundances which was not matched by any of the pigments. An explanation for these discrepancies could be due to differences in the relative abundance of individual cyanopigments observed in different cyanobacteria [35]. A recent study suggested that using the sum of the cyanopigments could provide a better representation of general cyanobacterial abundance in sediment core samples [36], but this has not yet been tested in comparison to sedDNA molecular analyses. Discrepancies could also reflect differences in degradation rates since DNA and pigments are very different molecules. Further detailed studies with the two methodologies applied in parallel are required to understand these observed discrepancies, and to gain new insights into the complementarity of these methods.

The present study compared a droplet digital PCR (ddPCR) assay, targeting a cyanobacteria-specific region of the 16S rRNA gene, with high-performance liquid chromatography (HPLC) focusing on the concentrations of four pigments largely confined to cyanobacteria (cyanopigments—canthaxanthin, echinenone, myxoxanthophyll and zeaxanthin). Droplet digital PCR is a relatively recent technique that generates an emulsion of approx. 20,000 separate PCR reactions as droplets in an oil solution. The target DNA sequence is amplified as in standard PCRs and then the number of DNA copies in each droplet is counted using fluorescence. It has numerous advantages over traditional qPCR, for example no inhibition assays or standard curves are required, and samples do not need to be run in triplicate since every result is an average of all the individual PCR reactions corrected by a Poisson distribution. Pigment analysis by HPLC separates a complex mixture of compounds in samples (which have been extracted and concentrated) based on the interaction of individual compound species with an adsorbent material (the column packing) and a gradient of solvent flowing through the column. A photo-diode array (PDA) detector then gives a quantitative measure of each component as they elute from the column, based on their light-absorbance pattern.

In this study, historical cyanobacteria abundances were determined using these two techniques and compared in 132 samples taken from four lake sediment cores, spanning periods of ca. 700 to 900 years. We hypothesized that: (1) there would be strong correlations between cyanobacteria-specific 16S rRNA gene copies (determined by ddPCR) and cyanopigments (determined by HPLC) across all lakes and core depths, and (2) the sum of all cyanopigments would have stronger relationships to cyanobacterial 16S rRNA gene copy numbers compared to individual pigments.

## 2. Materials and Methods

### 2.1. Study Sites and Sediment Core Sampling

Four lakes were sampled between September 2018 and July 2019 in the North Island of New Zealand: Lakes Nganoke, Okataina, Pounui, and Rototoa. These lakes were selected because they have good sedimentary records and different contemporary cyanobacterial abundances: Lakes Nganoke and Pounui experience cyanobacterial blooms every summer, while Lakes Rototoa and Okataina are deeper lakes with no cyanobacterial blooms (Table S1). Sediment cores were retrieved from a site closest to the deepest point of each lake using an Uwitec gravity corer (UWITEC, Mondsee, Austria) and 2 m-long 90-mm diameter polyvinyl chloride barrels (Leda Extrusions NZ ltd, Upper Hutt, New Zealand). All core barrels were cleaned with 2% sodium hypochlorite (bleach) prior to coring. After retrieval, the cores were sealed and stored at 4 °C in darkness for up to 4 weeks until sub-sampling.

The Nganoke core was 91 cm long which corresponds to ca. 1130 AD [37], the Ro-totoa core was 75 cm long (ca. 1690 AD, unpublished data Table S2), the Okataina core was 124 cm long (ca. 1720 AD [38]) and the Pounui core was 73 cm, dating to ca. 1280 AD [39]. The sediment cores were split in half using a using a bench mounted Geotek core splitter (Geotek Ltd., Deventry, Northamptonshire, UK) in a dedicated room (no molecular analysis). Due to the guillotine smearing the sediment upon core splitting, 2–3 mm from the surface of one half-core were carefully removed with a large, sterilized spatula (dipped in ethanol and blow-torched). Sediment was subsampled (2–3 g) from the center of the half-core using a sterile plastic scoop or a sterilized spatula at various depths down the cores and frozen (−20 °C) until further use.

### 2.2. Cyanobacteria Primers Modification

Partial 16S rRNA cyanobacteria sequences of all main types of cyanobacteria (picocyanobacteria, benthic, pelagic bloom-forming) and some non-target bacteria were selected from GeneBank and aligned (140 sequences in total) with MEGAX (Pennsylvania State University, State College, PA, USA) [40]. This alignment was assembled to assess whether primers CYAN108F and CYAN377R [41] adequately amplified all cyanobacteria (a selection of the alignment is shown in Figure S1). This in-silico analysis revealed that picocyanobacteria such as *Cyanobium gracile* and some *Synechoccocus* had a one-nucleotide difference with CYAN108F and all cyanobacteria species in the alignment had a one-nucleotide difference with CYAN377R (Figure S1). Both primers were therefore slightly modified to enhance the amplification of as many cyanobacteria as possible while not amplifying other non-target bacteria (Table 1). The specificity towards cyanobacteria was further checked in-silico by running both primer sets (old and new) with TestPrime (v 1.0, Ribocon GmbH, Bremen, Germany) [37], which runs an in-silico analysis using the SILVA 16S database.

### 2.3. Water Content Determination

Sediment core samples were thawed in the dark at 4 °C, subsamples (0.5–1 g) were weighed into pre-weighed glass vials, lyophilized (Gamma 1–16 LSC freeze-dryer; Martin Christ Gefriertrocknungsanlagen, Osterode am Harz, Germany), and re-weighed. The water content was determined using the following formula:

(1) $$\mathrm{Water}\;\mathrm{Content}\;\mathrm{of}\;\mathrm{sediment}=\frac{\mathrm{Wet}\;\mathrm{Weight}\;\left(g\right)-\mathrm{Dry}\;\mathrm{Weight}\;\left(g\right)}{\mathrm{Wet}\;\mathrm{Weight}\;\left(g\right)}$$

### 2.4. DNA Extraction and Droplet Digital PCR

The molecular analysis was conducted sequentially in separate sterile laboratories dedicated to each step (DNA extraction, ddPCR set-up, template addition, and PCR amplification) to ensure no cross-contamination. Each room (except for PCR amplification) was equipped with ultra-violet lights on the ceiling for sterilization, switched on for 40 min before and after each use. Furthermore, ddPCR setup and template addition were undertaken in laminar flow cabinets, with HEPA filtration and 15 min UV sterilization before and after use. Aerosol barrier tips (epT.I.P.S., Eppendorf, Hamburg, Germany) were used throughout.

The DNeasy PowerSoilTM DNA Isolation Kit (QIAGEN, Hilden, Germany) was used for sedDNA extraction. Approximately 0.25 g of wet sediment was weighed in the first tube of the kit (bead tube) and exact weights for each subsample recorded. DNA extraction was performed in batches of 12 samples including a negative control every two batches (all the reagents but no sediment) using a QIAcube (QIAGEN, Hilden, Germany) for automated extraction following the manufacturer’s protocol.

Following extraction, cyanobacterial 16S rRNA gene copy numbers were quantified using ddPCR. All samples had to be diluted for adequate quantification; dilution ranged from 1/10 to 1/10,000. The ddPCR workflow was undertaken using a BioRad QX200 system (Bio-Rad laboratories, Hercules, California, United States) following the manufacturer’s protocol and the methods described in a previous paper [42]. Due to the change in primers, the annealing temperature was lowered to 55 °C for 1 min (full Mastermix composition and cycling conditions in Table S3). The Lake Nganoke sedDNA samples were analysed using the CYAN108F and CYAN377R (Table 1) and the primers designed in this study to compare levels of amplification. Cyanobacteria concentration obtained with the QuantaSoft software were then standardized to DNA gene copy numbers per gram of dry sediment using the following formula:

(2) $$\mathrm{Gene}\;\mathrm{copies}\;=\frac{\mathrm{ddPCR}\times \frac{22\;µL}{4\;µL\;}\times \mathrm{DF}\times 100\;µL}{\mathrm{sed}.\;\mathrm{weight}\times \left(1-\;\mathrm{water}\;\mathrm{content}\right)}$$

where gene copies = cyanobacteria (16S rRNA) gene concentrations (gene copies/gram of dry sediment), ddPCR = concentration of 16S rRNA gene copies per µL, 22 µL was the volume of the MasterMix used, 4 µL was the volume of DNA template added to the PCR reaction, DF = dilution factor (10 to 10,000), 100 µL was the volume the DNA was eluted in during extractions, sed. weight = exact weight of each subsample extracted for DNA (~0.25 g), and water content = water content of the core subsample (from Equation (1)).

The limit of quantification (LoQ) of the new cyanobacterial 16S rRNA primer set was determined using a dilution series of a positive control (DNA extracted from a cyanobacterial-dominated mat) and a negative control (extraction using the PowerSoil kit with no sample added), in duplicate. The LoQ was determined as the cyanobacterial 16S rRNA gene concentration of the lowest dilution where a linear relationship was still measured. At this point there was a clear distinction between positive droplets in the positive control and the cloud of negative droplets observed in the negative control.

### 2.5. Pigment Extraction and High-Performance Liquid Chromatography

Sediment core samples were thawed in the dark at 4 °C, and subsamples (0.5–1.2 g) were extracted three times using acetone and ultrasonication (30 min) in a bath sonicator (Kudos Ultrasonic Cleaner; Shanghai, China) with ice. The extract was dried under a stream of nitrogen gas at 40 °C and stored at −20 °C until analysis. On the day of analysis, the dried extract was resuspended in acetone (0.5 mL) and transferred to a septum-capped amber vial for analysis by HPLC with DAD using an Agilent 1260 system (Santa Clara, CA, USA). Pigments were separated using a C_30_ column (Develosil RP-Aqueous C_30_, 5-µm, 250 × 4.6 mm; Phenomenex, Torrance, CA, USA) maintained at 30 °C and a gradient of methanol + 0.1% triethylamine (Solvent A) to 40:60 methanol/isopropyl alcohol + 0.1% triethylamine (Solvent B). Samples were injected (10 µL injection) in 100% Solvent A, which was maintained for 5 min before proceeding in a linear gradient to 65% Solvent B over 35 min. The column was washed with 90% Solvent B for 5 min and re-equilibrated in 100% Solvent A for 5 min between each injection. The flow rate was 1 mL/min throughout the chromatographic gradient. Light absorption data were collected over a 320–800 nm wavelength range, but only specific wavelength ranges were used for compound quantitation during postprocessing (Table 2).

A five-point mixed standard curve (0.5–20 µg/mL) of lutein (Carotenature, Münsingen, Switzerland) was analyzed with each HPLC run along with qualitative standards for each pigment analysed. The lutein standard was calibrated by spectrophotometry at 445 nm using the extinction coefficients described in Roy et al., (2011) [43]. Equivalence factors for canthaxanthin, echinenone, myxoxanthophyll and zeaxanthin (Table 2) were determined in relation to lutein by analyzing standards at known concentrations. These equivalence factors were used for the routine quantification of the other pigments, rather than preparing a standard curve for each HPLC run.

### 2.6. Data Analysis

Analysis was undertaken using R software (v4.0.2, Vienna, Austria) [44] and RStudio software (v1.3.959, Boston, MA, USA) [45]. Data manipulation, exploration, and plots were made with Tidyverse (v1.3.1) and associated packages [46].

Differences in 16S rRNA gene copy quantification between the previous PCR primer and the newly developed primer set were investigated using the Lake Nganoke sediment core. Cyanobacterial concentrations obtained from the ddPCR using both primers were first plotted as a down-core profile, and then summarized using paired boxplots. Log10 transformation was undertaken, and a paired *t*-test was performed on the transformed data to evaluate whether the new primer set amplified a high number of cyanobacterial 16S rRNA gene copies.

Total cyanopigments were calculated from the sum of the concentrations of each cyanobacterial pigment per lake. All proxies were first visualized as down-core profiles to look at general trends. Last, cyanobacterial 16S rRNA gene copy concentrations from ddPCR data were then compared to cyanopigment concentrations from HPLC using scatterplots and Spearman’s correlation. Four samples from the tephra in-wash in Lake Okataina had very low 16S rRNA gene copies levels (lower than the LoQ) and were not compared with cyanopigments. The subsample depths for each core were visualized using colour codes. Differences in correlations to pigments depending on the primer set were represented using scatterplots (Lake Nganoke only).

## 3. Results

### 3.1. Cyanobacterial 16S rRNA Gene Primer Comparison

Modifying the cyanobacteria-specific 16S rRNA ddPCR primers increased the groups of cyanobacteria detected, as revealed by both the in-silico analysis and sediment core test. The in-silico analysis using TestPrime only found two matches for the CYAN108F and CYAN377R primer set when no nucleotide mismatch was allowed: a species of *Calothrix* (cf. *Calothrix sp.* ‘muscicolous cyanobiont 5’) as well as one sequence of chloroplast (unclassified). The new primer set CYAN107F and CYAN377R_mod amplified (in-silico, no mismatch) 3842 cyanobacteria sequences of the 4700 (82%) in the SILVA database, including 223 genera of photosynthetic cyanobacteria (oxyphotobacteria). Most importantly for our study, the new primer set amplified specific genera found in New Zealand lakes, including the most abundant taxa identified a recent study [42]: *Aphanizomenon, Cyanobium, Dolichospermum, Microcystis, Nodosilinea,* and *Tychonema* (potentially not amplified by the previous primer set). The in-silico analysis indicated that the new primer set CYAN107F and CYAN377R_mod also amplified some nontarget bacteria and chloroplasts sequences (respectively 60 and 454 sequences), since the amplicon targets a conserved region of the 16S rRNA gene. Plastids from some diatoms could be potentially co-amplified, other plastids were from plants and algae that are non-native to New Zealand and very unlikely to be found in and around the study lakes. Overall, twenty-six plastids were amplified in-silico by the new primer set compared to the original primer set (Table S4).

**Figure 1:**
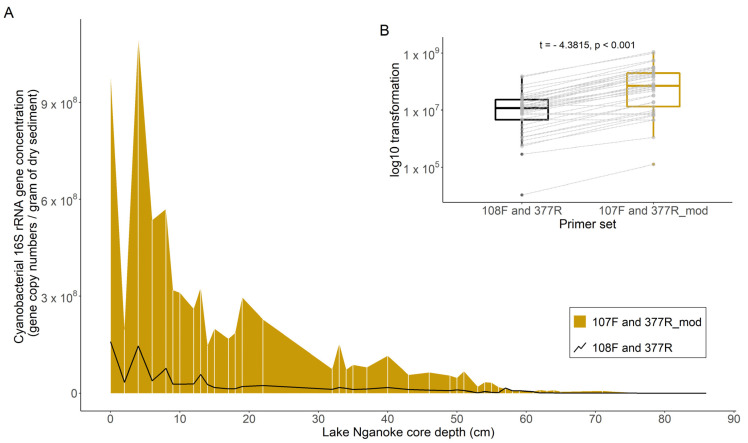
(**A**) Differences in cyanobacterial 16S ribosomal RNA gene copy numbers between the primer set CYAN108F and CYAN377R [41] and the primer set developed in this study (CYAN107F and CYAN377R_mod) when applied to sedimentary DNA extracted from a sediment core from Lake Nganoke. White vertical lines show sample depths. (**B**) A paired t-test was run on the log-transformed data and the results are displayed in the inset. The y-axis is the same as for (**A**), cyanobacterial 16S rRNA gene concentration, but with a log10 transformation.

The sedDNA from Lake Nganoke was used to compare the two primer sets. The concentrations of 16S rRNA gene copies were on average 7.8-times higher with the new primer set (CYAN107F and CYAN377R_mod; Figure 1A), and a paired *t*-test showed this was a significant difference (*p*-value < 0.01, Figure 1B). The downcore profile showed that both primer sets reproduced similar trends overall. For example, there were some common peaks at depths of 0, 4, 13, 19, 33, and 40 cm, but the number of gene copies detected with the new primer set was markedly higher from 55 cm onwards (ca. 1561 AD). Furthermore, when the concentrations of the cyanobacterial 16S rRNA gene (from both primers) were compared to the concentrations of individual cyanopigments also found in the Lake Nganoke sediment core (canthaxanthin, echinenone, myxoxanthophyll and zeaxanthin), stronger relationships were observed using the new primer set (0.65 ≤ R^2^ ≤ 0.81 for the new primer set, 0.59 ≤ R^2^ ≤ 0.72 for the original primer set; Figure S2). The LoQ for the new primer set (CYAN107F and CYAN377R_mod) was 0.4 16S rRNA gene copies per µL (raw ddPCR value) using DNA extracted from the positive control. When normalized to the standard sediment weight (0.25 g) and the mean water content (70%) using the formula described in the Methods section (Section 2.4), the LoQ was 2933 16S rRNA gene copies per gram of dry sediment.

### 3.2. Cyanobacterial 16S rRNA Genes in Sediment Cores

**Figure 2:**
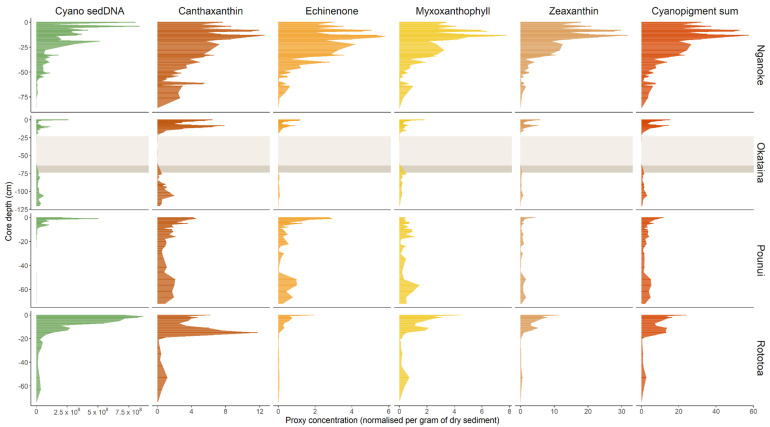
Cyanobacterial 16S rRNA gene, individual, and total cyanopigments downcore profiles for each lake. Cyano sedDNA = Cyanobacterial 16S rRNA gene copy numbers per gram of dry sediment, cyanopigment concentration (µg per gram) of dry sediment. Cyanopigment sum refers to the sum of the four individual pigments. The grey shades in Lake Okataina indicate the tephra (dark) from the 1886 Mount Tarawera eruption and tephra in-wash (light) from the catchment, which affected the detection of cyanobacteria.

Cyanobacterial 16S rRNA genes were successfully detected in every sample analyzed (some at very low levels–minimum 255 copies/g dw). For all lakes, higher cyanobacterial 16S rRNA concentrations were observed at the top of the cores and decreased in the older sediment samples (Figure 2). The exception to this was Lake Okataina, where the concentrations observed at the top of the core were lower than other lakes, and levels were relatively stable over the length of the core. Furthermore, the cyanobacterial 16S rRNA gene copy levels measured above and below the tephra from the 1886 Mount Tarawera eruption were similar (Figure 2). The highest concentrations of 16S rRNA gene copies were detected in Lakes Rototoa and Nganoke (~8.5 × 10^8^), while Lakes Pounui and Okataina showed lower levels (Figure 2). There was a substantial nine-fold difference in mean cyanobacterial 16S rRNA gene copy levels between Lake Rototoa and Lake Okataina.

### 3.3. Cyanobacterial Pigments

Cyanopigment concentrations were variable across lakes and within the same core; some high pigment concentrations were observed at the bottom of the cores, especially canthaxanthin in all lakes, and all cyanopigments in Lake Pounui (Figure 2 and Figure S3). Zeaxanthin was generally the most abundant cyanopigment across all lakes (max. 32 µg/g dw), followed by canthaxanthin (max. 13 µg/g dw), while myxoxanthophyll and echinenone displayed the lowest levels (max. ~6 µg/g dw, respectively). The sum of all four cyanopigments (total cyanopigments) reflected individual pigment trends and was overall highest in Lake Nganoke (mean = 17.5 µg/g dw) and lower in Lakes Okataina, Pounui, and Rototoa (mean = 3.4, 3.6, and 7.1 µg/g dw, respectively). Zeaxanthin and canthaxanthin made the greatest contribution to total cyanopigments (max. 60% and 80%, respectively), while myxoxanthophyll and echinenone had lower contributions (max. 34% and 11%, respectively) (Figure 2, Figures S3 and S4). Individual cyanopigments were not detected in every sample; for example, in Lake Okataina some layers of tephra in-wash (volcanic ash washed in from the catchment) did not yield any cyanopigments, and there was no echinenone nor zeaxanthin detected in the oldest sample (93 cm core depth) of Lake Nganoke (Figure 2).

### 3.4. Proxy Relationships within Each Lake

**Figure 3:**
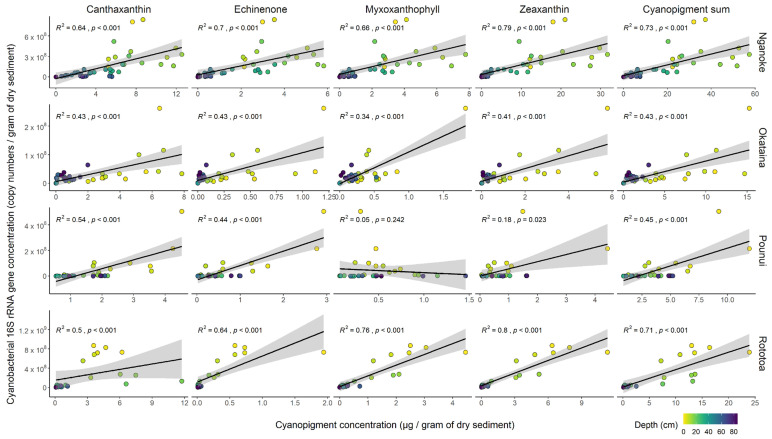
Relationships across lakes between cyanobacterial 16S rRNA gene concentrations (gene copy numbers per gram of dry weight of sediment) from droplet digital PCR data and concentrations of individual cyanopigments and total cyanopigments from high-performance liquid chromatography (µg per gram of dry weight of sediment). Relationships (R^2^) and *p*-values calculated using Spearman’s correlation test are displayed, colour gradient shows the depth of each core sub-sample.

The relationships between the cyanobacterial 16S rRNA gene concentration and the individual cyanopigments (canthaxanthin, echinenone, myxoxanthophyll and zeaxanthin) were variable. In Lake Nganoke, the Spearman’s correlation was strong for each individual cyanopigment (R^2^ ≥ 0.64, *p* < 0.001; Figure 3). In Lake Pounui, the best correlation was for canthaxanthin (positive, strong, and significant; R^2^ = 0.54, *p* < 0.001) and the worst correlation was for myxoxanthophyll (not significant, R^2^ = 0.05, *p* = 0.24). In Lakes Okataina and Rototoa, significant correlations were observed for each individual cyanopigment (*p* < 0.001), although the strength of the correlation varied (0.34 ≤ R^2^ ≤ 0.8). To overcome the differences observed between individual cyanopigments, the sum of all cyanopigments was compared with cyanobacterial 16S rRNA gene copy concentrations. Total cyanopigments were always positively and significantly correlated to 16S rRNA gene copies for all lakes (0.43 ≤ R^2^ ≤ 0.73, *p* < 0.001, Figure 3) and were more consistent than any individual cyanopigment. The correlation of total cyanopigments to 16S rRNA gene copies was the best-equal for Lake Okataina (alongside canthaxanthin and echinenone). In Lakes Nganoke and Pounui, total cyanopigments was second best (behind zeaxanthin and canthaxanthin, respectively). In Lake Rototoa, total cyanopigments had the third highest Spearman’s correlation coefficient, but this was close to that of the individual pigments demonstrating stronger relationships (R^2^ = 0.71 for total pigments, 0.76 for myxoxanthophyll, 0.8 for zeaxanthin).

## 4. Discussion

### 4.1. Cyanobacterial-Specific Primer Design and Testing

Initial explorations suggested that the original cyanobacterial 16S rRNA primer set (CYAN108F and CYAN377R [41]) was unlikely to amplify a wide range of cyanobacteria in the sediment samples when no mismatches were allowed. These primers were first designed more than 15 years ago. Since then, there has been a rapid increase in the number of 16S rRNA sequences available in databases and this allowed us to enhance the specificity of PCR primers for this target. The in-silico analysis indicated that with no mismatches allowed, the primer set CYAN108F and CYAN377R would be unlikely to amplify the picocyanobacteria known to occur in New Zealand lakes [42]. While other cyanobacterial 16S rRNA gene qPCR primers have been developed [47], we were unable to adapt these to the ddPCR workflow used in this study, because positive droplets did not segregate away from negative droplets. Therefore, slight modifications were made to the CYAN108F/CYAN377R primer set to enable the amplification of a wider range of cyanobacteria. The amplicon from the new primers was slightly longer than recommended for ddPCR (283 bp vs. <200 bp [48]), which resulted in some noise (intermediate droplets between positive and negative droplets) when the samples were too concentrated. However, upon adequate dilution the positive droplets segregated correctly.

The new primers (CYAN107F and CYAN377R_mod) resulted in cyanobacterial 16S rRNA gene concentrations that were on average 7.8-fold higher compared to the original primer set. This was most likely because the new primer set detected a wider range of cyanobacteria taxa, which is of particular value for paleolimnological studies. This allowed an increase in cyanobacteria to be detected from ca. 1560 AD up to present time in the Lake Nganoke sediment core, whereas the previous primer set would have detected an increase much later (ca. 1850s). The downcore 16S rRNA gene profiles produced with the new primer set also yielded a stronger fit to historical cyanopigment concentrations compared to the original primer set.

### 4.2. Correlation between Cyanobacterial 16S Ribosomal RNA Gene Copies and Cyanopigments

In general, there were moderately strong positive relationships between the cyanobacterial 16S rRNA gene and cyanopigment concentrations but differences among lakes. A recent study evaluated a range of cyanobacteria species and reported differences in the relative concentrations of individual cyanopigments [36]. In particular, picocyanobacteria (Synechococcaceae) contained lower relative concentrations of echinenone than other cyanobacteria, and bloom-forming genera such as *Dolichospermum* and *Microcystis* contained lower relative concentrations of zeaxanthin. Differences in the cyanobacterial community over time were evident in Lake Pounui, where echinenone was more abundant than myxoxanthophyll in the top of the core, and in Lake Rototoa where myxoxanthophyll was more abundant from the middle of the core. These types of shifts in specific cyanopigments have been well documented in other lakes in the literature [21,24,25] and have been theorized to be associated with changes in the cyanobacterial community.

Pal et al. (2015) also applied cyanobacteria-specific qPCR primers and pigment analysis to five sediment cores from Western Quebec (Canada). The relationships between DNA and pigment analyses (echinenone and zeaxanthin) showed high between-lake variability, with some positive and some negative correlations. Although no explanation was provided for this, it could be because only two ‘cyanopigments’ were targeted, because of the primers used (CYAN108F and CYAN377R; which may not detect all taxa), or due to the use of qPCR (which is more susceptible to inhibition). In contrast, Hobbs et al. (2021) found no pigment patterns to mirror cyanobacterial 16S rRNA gene copy numbers in a core from a eutrophic lake in Washington (USA), particularly at the top of the core where copy numbers increased in the absence of pigment increase. Reasons for the discrepancy are unknown, although we note that a different primer set (CyanoReal16S) was used for these ddPCR analyses. Because of the limited number of studies currently conducted in this area, using a variety of primers and pigment choices, it is currently difficult to comprehensively explain the different results observed between these studies and the current study.

### 4.3. The Sum of All Cyanopigments—A Better Proxy for Total Cyanobacterial Biomass?

As mentioned above, different cyanobacterial taxa contain varying relative abundances of canthaxanthin, echinenone, myxoxanthophyll, and zeaxanthin. A recent study analysed pigments in 34 cyanobacterial cultures and found, for example, that strains of *Dolichospermum* sp., *Nodularia spumigena*, and *Cuspidothrix issatchenkoi* produced high levels of canthaxanthin, while *Planktothrix* sp. produced more myxoxanthophyll than other cyanobacteria [36]. Furthermore, myxoxanthophyll was the only pigment not detected in all (10 out of 34) studied cultures; it was only detected in all of the picocyanobacteria (Synechococcaceae), as well as *Planktothrix* sp. and one *Microcoleus autumnalis* culture. For this reason, if only one or a few cyanopigments are analysed, a marked portion of cyanobacterial biomass may go unaccounted for. In the present dataset, this may partially explain why the strongest relationship between individual cyanopigment concentrations and 16S rRNA gene concentrations varied across lakes. To overcome these biases and to draw inferences on the whole cyanobacteria community, we propose that future studies should analyse the suite of cyanobacterial pigments presented here (or a broader suite) and evaluate the sum of these pigment concentrations.

When analysed individually or in combination with one another, the pigments may also provide some information on the composition of the cyanobacterial community. For example, a recent study showed that ratios of selected pigments could be used to assess the relative abundance of picocyanobacteria and bloom-forming cyanobacteria in a sample [36]. Given that these data are required when calculating the total cyanopigments values, it allows multiple levels of information on the cyanobacterial community to be obtained from the HPLC pigment analysis.

### 4.4. Reasons for Discrepancies between the Two Methods

As noted above, the relationship between cyanobacterial 16S rRNA gene copy concentrations and individual cyanopigment concentrations varied across lakes and were not always strong. There are many plausible reasons for these discrepancies, primarily related to the fundamental difference in what each technique measures. The ddPCR assay measures the number of copies of the cyanobacteria-specific 16S rRNA gene in a sample, akin to a traditional microscopic cell count. However, some cyanobacteria can have multiple copies of the 16S rRNA gene within their genomes (e.g., two copies in *Microcystis* and picocyanobacteria such as *Synechococcus*, *Synechocystis*, and four copies in *Nostoc*; [49,50]); therefore, in these instances cyanobacterial abundance will be overestimated. Using an approach where ddPCR is coupled with a community characterization technique such as metabarcoding may help in establishing periods where taxa with multiple 16S rRNA gene operons exist, allowing data to be normalized. However, this would be time-consuming and challenging because the exact number of 16S rRNA gene operons is not known for all species. Challenges linked to the 16S rRNA gene such as non-specificity and multiple copy numbers could be solved by targeting other genes such as those involved in pigment production, which are generally single copy. However, this would not solve other issues previously mentioned, such as the lack of databases, which would be required for designing universal primers. Besides, in contrast to gene copy numbers, pigment quotas are affected by cell size, irradiance, and nutritional status, in addition to species-specific characteristics [51]. In the same way that ddPCR is close to an estimate of cell numbers, pigments are more likely to approximate biomass, albeit modulated by growth conditions and taxonomy. For example, two recent studies indicated that the cyanobacterial communities in at least some of the study lakes included large numbers of picocyanobacteria [36,42]. These are very small (<1 µm) and can be highly abundant even in oligotrophic lakes [52,53]. The ddPCR assay will give an even weight (provided they have the same number of 16S rRNA gene copies) to a picocyanobacteria cell as to a larger non-picocyanobacteria cell, whereas pigments will not.

A further reason for discrepancies between the two methods is that neither is truly cyanobacteria-specific. It is likely that the ddPCR primers are amplifying nontarget sequences, such as DNA from chloroplasts, while potentially missing some cyanobacteria. Likewise, some ‘cyanopigments’ are produced by organisms that are not cyanobacteria (e.g., zeaxanthin is known to be produced by some eukaryotic microalgae [51]). Furthermore, the relationships between the two proxies were most variable near the top of the sediment cores. Both pigments and DNA are susceptible to degradation, and this is most evident at the top of sediment cores [54] where sharp declines in both proxies were observed in our data. However, the bottom of the Lake Okataina core had the highest 16S rRNA gene copies levels compared to the other cores, but the pigments show a different trend across lakes. This could be due to age differences across sediment cores, since the bottom of the Okataina core was younger than other cores (ca. 1700s in Lake Okataina vs. ca. 1300s or older in Lakes Nganoke and Pounui [37,38,39], Table S2). It is also likely that the rates of degradation vary between DNA and pigments, which would explain the discrepancies observed in downcore profiles. A strategy that could be used to minimize the impact of degradation on the results and is commonly applied in paleolimnological analysis is the normalization of the data across samples, for example to total DNA, chlorophyll-a, or total pigments. Given that the aim of this study was to directly compare the methods in terms of concentration of cyanobacteria per sample, this was not undertaken here. A final consideration is that lake sediments vary in the degree and types of inorganic and organic material they contain, and this may change down a sediment core as it reflects changes in the landscape around a lake. The presence of some compounds, for example, humic acid, can make extracting high-quality DNA and pigments challenging. For example, Pal et al. (2015) were unable to detect zeaxanthin and echinenone in a lake sediment core and proposed it could be due to the high humic acid content of the sediment core [22].

### 4.5. Comparing the Pros and Cons of ddPCR and HPLC to Track Historic Cyanobacteria Abundances

Pigment analysis using HPLC has been used for many decades in paleolimnological studies [8,9,10,11,12], providing a wealth of comparative data. In contrast, ddPCR is a relatively new method [55,56], and a limited number of studies have used cyanobacteria-specific ddPCR assays. As discussed above, methodological choices, such as primer sets and data normalization, need optimizing. Evaluation of the relative value of these two techniques is thus based on different amounts of information.

Capital expenditure for both techniques is relatively high (~USD 57,000 /EUR 48,000 for HPLC systems and ~$121,000 USD/102,000 € for the QX200 automated ddPCR workflow in 2021). Robotics can be used during the DNA extraction step, making this process markedly quicker and more cost/time efficient than pigment extraction. Analysis of ddPCR samples is also quicker, with a batch of up to 96 samples being run in about four hours compared to HPLC which may only be able to analyse one sample per hour (depending on the chromatography adopted). Whilst ultra-performance liquid chromatography (UPLC) systems can be used to reduce the analysis times for cyanopigments, we have found that UPLC systems cannot achieve the required sensitivity and separation-efficiency for complex sediment extracts. Based on our analysis, including technician time, the cost per sample is approximately seven times cheaper on the ddPCR compared to HPLC when a batch of more than 20 samples is processed.

As noted above the two methods provide different information on cyanobacteria, 16S rRNA gene copies is a proxy for cell density and pigments for biomass, and each comes with caveats that need to be considered during interpretation. An advantage of ddPCR is that primers can target any gene of interest provided there is prior knowledge on its sequence, and multiple assays can be undertaken in one run (multiplexed) with limited additional costs. For example, to provide insights into the composition of the cyanobacterial community or target problematic species, multiplexed ddPCR assays could have up to four targets (QX200 system) or more targets (modern ddPCR systems becoming available now). Potential targets could be total cyanobacteria (using the cyanobacterial 16S rRNA gene as described here), specific cyanobacteria species or strains (by targeting more variable portions of the 16S rRNA gene or the intergenic-spacer sequence of the 16S and 23S rRNA genes), or cyanotoxin production (by targeting toxin production genes). Both ddPCR and HPLC require a high level of expertise to establish the assay on the user’s system, and once set up a high level of laboratory skill is needed to ensure robust results. This study has demonstrated that both methods provide similar patterns in terms of difference among and within lakes in terms of cyanobacterial abundance. The pros and cons of each method (above and summarised in Table 3) should be considered when selecting which method is more appropriate for the specific aims and scopes of the paleolimnological study.

## 5. Conclusions

This study reported moderately strong correlations between cyanobacteria-specific 16S rRNA gene copies (determined by ddPCR) and four pigments (determined by HPLC) commonly used as cyanobacteria-specific markers in lake sediment cores. Positive relationships between these two analyses were detected, and the relationships were more consistent between lakes when all pigments were summed rather than considered separately. From an ecological perspective, and given that the ddPCR assay detects all cyanobacteria, using a sum of all pigments was more logical and increased the likelihood that a range of different species were detected. Variability of the relationship among lakes and down sediment core depths were evident, which are likely related to the composition of the cyanobacterial communities in each lake and the nuances of each analytical method. The two methods tested here provide proxies for two different cyanobacterial abundance measures: cell density (cyanobacterial 16S rRNA gene—ddPCR) and biomass (pigments—HPLC). It is important that this is acknowledged and that the caveats of each method are taken into consideration during method selection and interpretation of the results. When applied to sediment cores, ddPCR analyses would ideally be undertaken in parallel with cyanobacteria metabarcoding (e.g., [42]) as this would provide a powerful approach to explore historic shifts in cyanobacterial communities alongside robust evaluation of cyanobacterial abundance through time.

## Figures and Tables

**Table 1:** Details of the modifications to the CYAN cyanobacteria primer set targeting a region of the 16S ribosomal RNA gene. Nucleotides added or modified are in bold. Total amplicon length is ~283 bp.

Primer	Sequence	Source
CYAN108F	5′-ACGGGTGAGTAACRCGTRA-3′	[41]
CYAN107F	5′-**G**ACGGGTGAGTAACRCGTR**RG**-3’	This paper
CYAN377R	5′-CCATGGCGGAAAATTCCCC-3′	[41]
CYAN377R_mod	5’-CCAT**T**GCGGAAAATTCCCC-3’	This paper

**Table 2:** Parameters of the high-performance liquid chromatography analysis of carotenoid pigments in sediment core samples. RT stands for retention time (minutes) and the wavelength is the range (nanometres) used for pigment quantification.

Pigment	RT	Wavelength	Equivalence Factor
Lutein	9.6	435–455	-
Zeaxanthin	10.3	435–455	1.56
Myxoxanthophyll	12.7	460–480	1.26
Canthaxanthin	16.1	460–480	1.06
Echinenone	32.9	435–455	0.63

**Table 3:** Summary of the pros and cons of using droplet digital PCR (ddPCR) and high-performance liquid chromatography (HPLC) to infer historical abundance of cyanobacteria from lake sediment cores.

Method	Pros	Cons
HPLC	Comparable with several decades of previously reported data.	Medium capital investment. Long sample turnaround (1 week for extraction and analysis of a batch of approx. 20 samples). Limited insight into cyanobacterial community composition. High expertise level required. Pigment degradation limits interpretation of more recent portions of sediment cores. Potential inhibition due to co-extracted compounds.
ddPCR	High throughput (up to 96 samples in one run). Rapid sample turnaround (1 day to extract DNA and analyse samples). Cost effective when multiple samples analysed simultaneously. Can be targeted to specific genera or species and up to four assays can be assessed at the same time on one sample (tetraplexing). Cost effective when a batch of samples is processed (about seven-times cheaper per sample than HPLC). Not hindered by inhibitors in sample.	Relatively new technique especially in paleolimnological research, so little comparative data. High capital investment (twice the price of HPLC instrument). Potential amplification of nontarget organisms. Prior knowledge of sequences needed to design specific assays. Potential multiple gene copies in one genome, therefore, not truly quantitative.

## Data Availability

Not applicable.

## Supplementary Materials

The following supporting information can be downloaded at: https://www.mdpi.com/article/10.3390/microorganisms10020279/s1, Figure S1: Selection of a few sequences from the nucleotide alignment of the cyanobacterial 16S rRNA gene with the two primer sets; Figure S2: Correlations between the new and original primer sets (107F / 377R_mod and 108F / 377R, respectively) with individual and summed cyanopigments in Lake Nganoke; Figure S3: Stacked view of the individual cyanopigments to visualise the contribution of each cyanopigment to total cyanopigment. White horizontal lines indicate sub-samples depths for each lake sediment core.; Figure S4: Percent stacked barplots showing the relative contribution of individual pigments to total cyanopigments; Table S1: Lake details. All lakes were cored at their deepest point (depocenter); Table S2: Lake Rototoa age model. Some rapidly deposited layers were observed between 24.7–28.1 cm and 44.3–53.9 cm, therefore theses depths were removed from the age model; Table S3: Mastermix and cycling conditions for the 107F and 377R_mod primer set; Table S4: List and details of the new chloroplasts (plastids) detected by the new primer set compared to the original set. Run in-silico on the SILVA database, with 1 bp mismatch.
