# Supplementary material for: Molecular and Pigment Analyses Provide Comparative Results When Reconstructing Historic Cyanobacterial Abundances from Lake Sediment Cores
Source: Microorganisms. 2022 Jan 25;10(2):279. doi: 10.3390/microorganisms10020279 (PMC8876145; doi:10.3390/microorganisms10020279)
Supplement: Supplementary file 1 [file microorganisms-10-00279-s001.zip › microorganisms-1524637-supplementary.pdf]

Supplementary Material

Table S1: Lake details. All lakes were cored at their deepest point (depocenter).

	Lake Nganoke	*Lake Okataina*	*Lake Pounui*	*Lake Rototoa*
Latitude	41° 21' 19" S	38° 6' 36" S	41° 20' 39" S	36° 30' 43" S
Longitude	175° 11' 13" E	176° 25' 17" E	175° 6' 48" E	174° 14' 19" E
Max depth (m)	2	78	9	26.1
Current water quality	Eutrophic	Oligotrophic	Eutrophic	Oligotrophic
Recent cyanobacterial blooms	Yes	No	Yes	No
Main land type in catchment	Pasture	Native / Pine forestry	Native	Pine forestry / Native

Table S2: Rototoa age model. Some rapidly deposited layers were observed between 24.7-28.1 cm and 44.3-53.9 cm, therefore theses depths were removed from the age model.

Depth (cm)	*Year (AD)*	*sigma*	*median*	*sed*	*from_95_4*	*to_95_4*
0	2018	0	2018	0.59	2017	2018
1	2016	0	2016	0.352	2016	2016
2	2012	0	2012	0.25	2012	2012
3	2008	0	2008	0.223	2007	2009
4	2003	0	2003	0.221	2002	2004
5	1999	1	1999	0.222	1998	2000
6	1994	1	1994	0.203	1993	1995
7	1989	1	1989	0.216	1988	1991
8	1985	1	1985	0.2	1983	1986
9	1979	1	1979	0.186	1977	1981
10	1974	1	1974	0.193	1972	1976
11	1969	1	1969	0.18	1967	1971
12	1963	1	1963	0.165	1960	1965
13	1957	1	1957	0.15	1954	1960
14	1950	2	1950	0.151	1947	1953
15	1944	2	1943	0.152	1940	1947
16	1936	2	1936	0.131	1933	1940
17	1928	2	1928	0.122	1924	1932
18	1920	2	1920	0.14	1916	1924
19	1914	2	1914	0.157	1910	1918
20	1907	3	1908	0.152	1899	1916
21	1901	4	1901	0.152	1893	1910
22	1894	4	1894	0.152	1885	1903
23	1888	3	1888	0.171	1878	1893
24	1882	4	1883	0.196	1872	1892
29	1874	5	1874	0.195	1863	1885
30	1869	5	1869	0.195	1857	1881

31	1864	6	1864	0.195	1851	1877
32	1859	6	1859	0.195	1846	1871
33	1854	6	1854	0.195	1840	1867
34	1849	7	1849	0.195	1834	1863
35	1844	7	1844	0.196	1829	1859
36	1838	7	1838	0.195	1823	1854
37	1833	8	1833	0.195	1817	1850
38	1828	8	1828	0.195	1812	1844
39	1823	8	1823	0.195	1806	1840
40	1818	8	1818	0.195	1801	1836
41	1813	9	1813	0.196	1795	1831
42	1808	9	1808	0.196	1789	1827
43	1803	9	1802	0.196	1784	1822
44	1797	9	1797	0.196	1779	1818
54	1795	9	1794	0.195	1776	1816
55	1790	9	1790	0.195	1772	1810
56	1785	10	1784	0.196	1765	1806
57	1780	10	1779	0.196	1760	1801
58	1774	10	1774	0.196	1754	1797
59	1769	10	1769	0.196	1749	1791
60	1764	10	1763	0.196	1744	1787
61	1759	11	1758	0.196	1739	1782
62	1754	11	1753	0.196	1733	1777
63	1749	11	1748	0.196	1728	1773
64	1744	11	1743	0.196	1723	1768
65	1739	11	1737	0.196	1718	1763
66	1734	11	1732	0.196	1713	1758
67	1729	11	1727	0.196	1707	1754
68	1723	12	1722	0.196	1702	1748
69	1718	12	1716	0.196	1697	1745
70	1713	12	1712	0.196	1693	1738
71	1708	12	1706	0.196	1687	1735
72	1703	12	1701	0.196	1683	1729
73	1698	12	1696	0.196	1676	1724
74	1693	12	1690	0.196	1673	1721
75	1688	12	1685	0.196	1667	1714

Table S3: Mastermix and cycling conditions for the 107F and 377R_mod primer set.

Primer set	*Master Mix (for one sample)*	*Cycling conditions*
107F and 377R_mod	10 μL Evagreen (2x)<br>7.6 μL DNA-free water<br>0.2 μL Forward primer (10 μM)<br>0.2 μL Reverse primer (10 μM)<br>4 μL template DNA	Initial denaturation: 95˚C - 5 minutes<br><br>50 cycles:<br>Denaturation: 95˚C - 30 seconds<br>Annealing: 55˚C - 1 minute<br><br>4˚C - 5 minutes<br>90˚C - 5 minutes

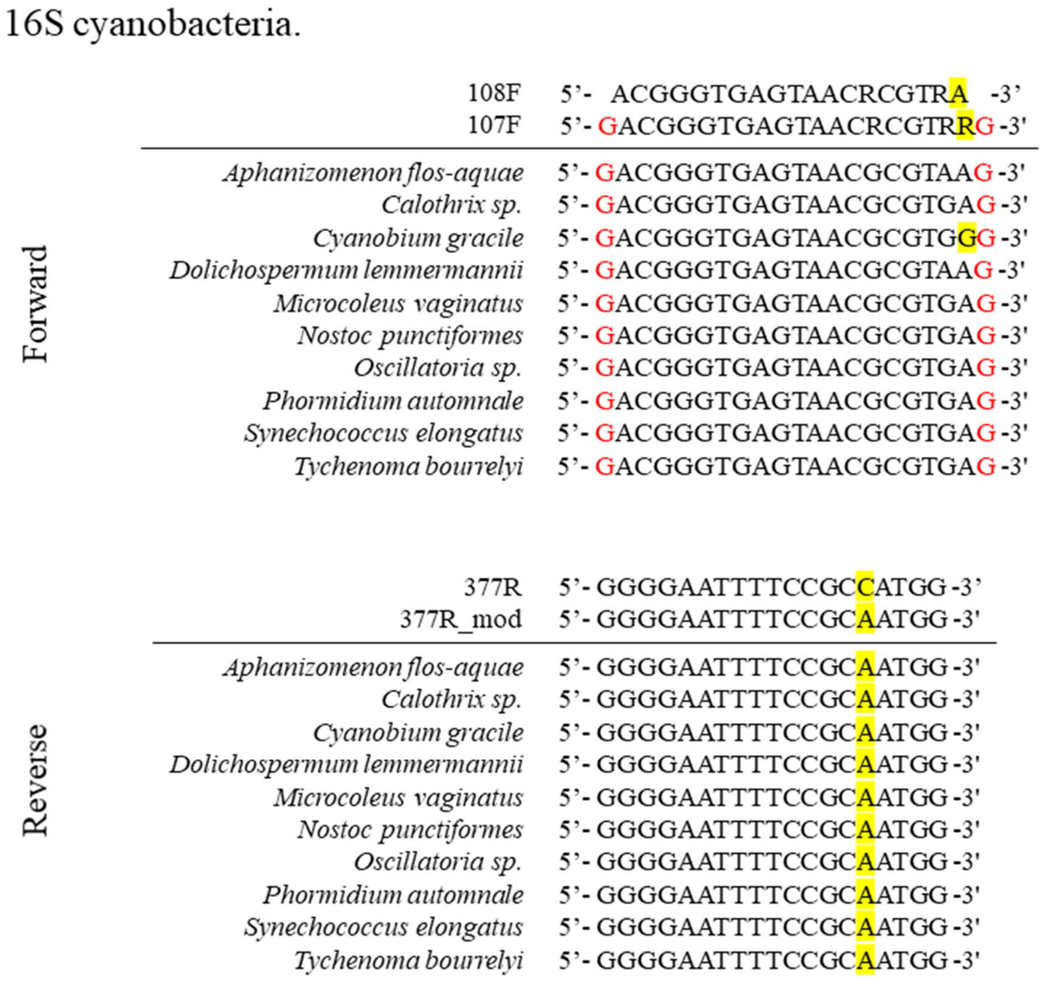

Figure S1: Partial nucleotide alignment of the cyanobacterial 16S rRNA gene with the two primer sets. The primer CYAN107F was improved from CYAN108F and CYAN377R_mod was improved from CYAN377R, both from Rinta-Kanto et al. (2005). Nucleotides highlighted in yellow show which nucleotides from the CYAN108F/CYAN377R primer set were modified in the 107R/CYAN377R_mod set. Nucleotides in red show the nucleotides added to the new forward primer.

Table S4: List and details of the new chloroplasts (plastids) detected by the new primer set compared to the original set. Run in-silico on the SILVA database, with 1 bp mismatch.

Scientific Name	*Common name*	*Found in*
Nephroselmis olivacea	green algae	freshwater
Zygnema circumcarinatum	charophycean alga	freshwater (not NZ)
Pelargonium x hortorum	zonal geranium	global
Austrotaxus spicata	New Caledonia yew / southern yew	New Caledonia
uncultured bacterium ARCTIC13_E_12		
uncultured bacterium ARCTIC24_F_09		
Micromonas commoda	green algae	marine environment
Bathycoccus prasinos	marine green alga	marine environment
Cephalotaxus hainanensis	Hainan plum-yew	Hainan peninsula (China)
Ecdeiocolea monostachya	tufted perennial	Western Australia
Passiflora ciliata	passionflower	natural range Mexico to North Colombia, South Florida to Caribbean
Wisteria floribunda	Japanese wisteria	Japan
Aquilaria sinensis	Aquilaria tree	China
Hafniomonas laevis	green algae	freshwater
Cylindrocystis brebissonii	green algae	freshwater
Thorea hispida	red algae	British Isles
Otohimella japonica	red algae	Japan (freshwater)
Oryza rufipogon	wild rice / red rice	East, South, Southeast Asia
Dermonema virens	red algae	marine environment
Izziella formosana	red algae	marine environment
Liagoropsis maxima	red algae	marine environment
Trichogloeopsis pedicellata	red algae	marine environment
Dichanthelium oligosanthes	Heller's rosette grass	North America
Polysiphonia schneideri	red algae	marine environment
Hemitomes congestum	Gnome plant	Western United States
Mantoniella squamata	green algae	marine environment

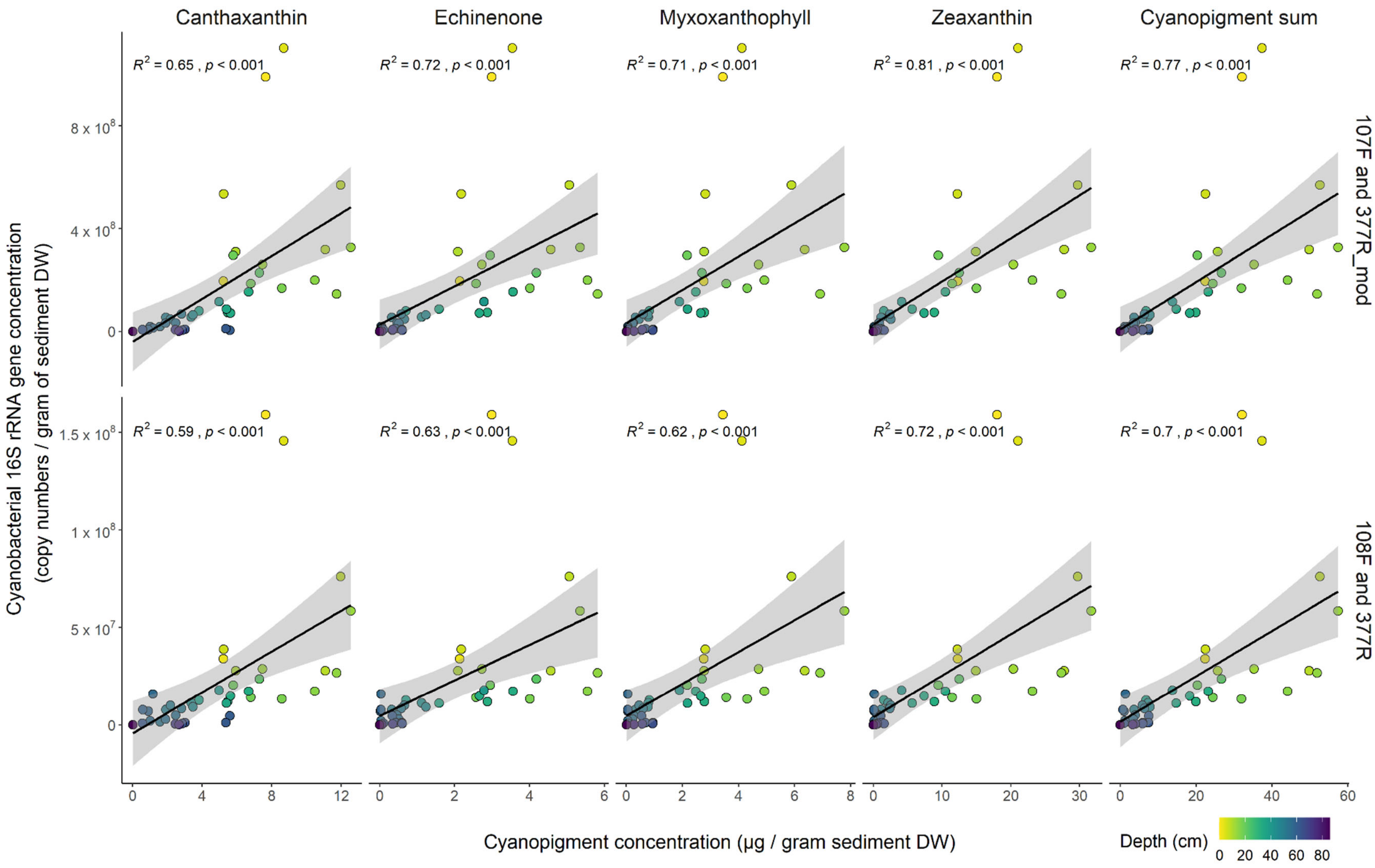

0

Figure S2: Correlations between the new and original primer sets (107F / 377R_mod and 108F / 377R, respectively) with individual and summed cyanopigments in Lake Nganoke. Spearman's correlations are displayed for each plot, DW stands for dry weight of sediment.

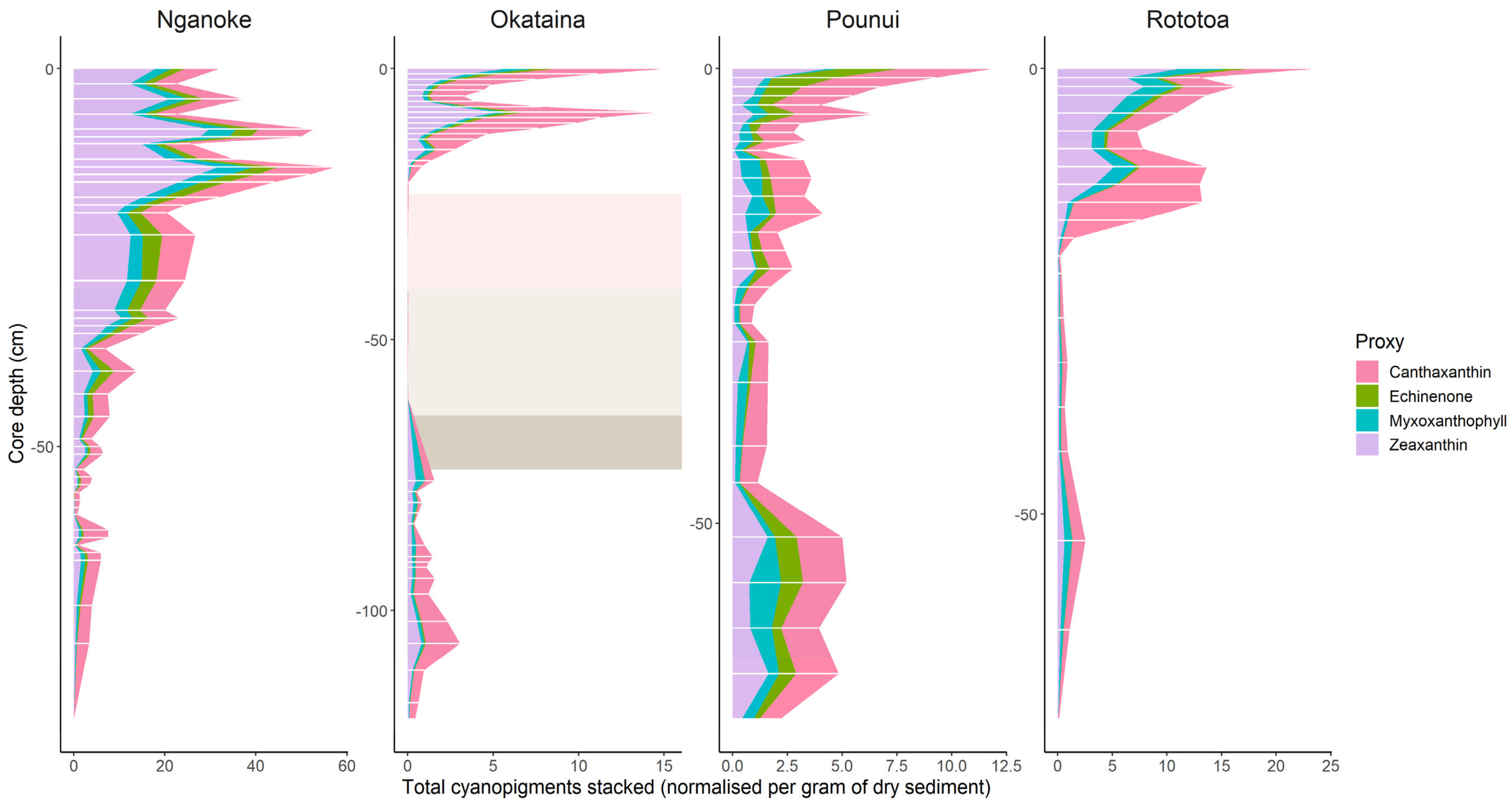

Figure S3: Stacked view of the individual cyanopigments to visualise the contribution of each cyanopigment to total cyanopigment. White horizontal lines indicate sub-samples depths for each lake sediment core.

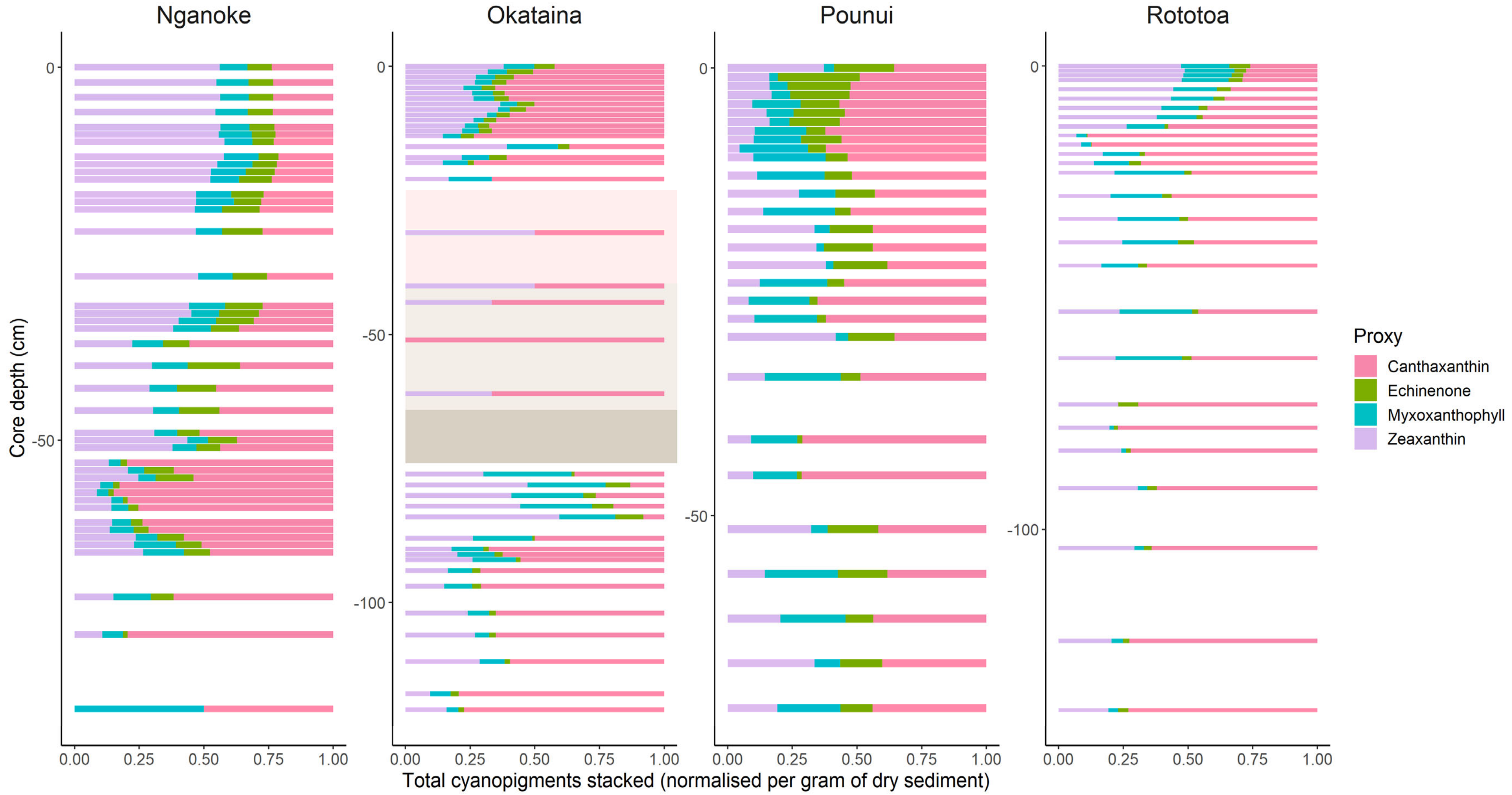

Figure S4: Percent stacked barplots showing the relative contribution of individual pigments to total cyanopigments.
